# “Acceptable” concentrations of enrofloxacin in food lead to reduced enrofloxacin susceptibility in a mouse model of gastrointestinal *Klebsiella pneumoniae*

**DOI:** 10.1128/spectrum.00385-25

**Published:** 2025-05-22

**Authors:** Zina Gestels, Bianca Torfs, Said Abdellati, Irith De Baetselier, Caroline Rombouts, Veronique Dermauw, Sheeba Santhini Manoharan-Basil, Chris Kenyon

**Affiliations:** 1 STI Unit, Department of Clinical Sciences, Institute of Tropical Medicine 37463 , Antwerp, Flanders, Belgium; 2 Applied Technology and Production unit, Institute of Tropical Medicine 37463 , Antwerp, Flanders, Belgium; 3 Clinical and Reference Laboratory, Department of Clinical Sciences, Institute of Tropical Medicine 37463 , Antwerp, Flanders, Belgium; 4 Unit of Zoonoses, Department of Biomedical Sciences, Institute of Tropical Medicine 37463 , Antwerp, Flanders, Belgium; 5 Division of Infectious Diseases and HIV Medicine, University of Cape Town 575383, Cape Town, Western Cape, South Africa; Central Texas Veterans Health Care System, Temple, Texas, USA; University of Veterinary Medicine, Budapest, Hungary

**Keywords:** minimum selection concentration, acceptable daily intake, ADI, *Klebsiella pneumoniae*, enrofloxacin, antimicrobial resistance

## Abstract

Concentrations of antimicrobials up to 1,000-fold lower than the minimum inhibitory concentration can select for antimicrobial resistance. This generates the hypothesis that the low concentrations of antimicrobials allowed in our food could select for resistance. We assessed if the dose of enrofloxacin allowed in food by the European Medicines Agency (6.2 µg/kg) could decrease susceptibility to enrofloxacin in a strain of *Klebsiella pneumoniae* colonizing the gastrointestinal tracts of Specific Opportunistic Pathogen-Free Naval Medical Research Institute (NMRI) mice. We found that one-tenth of this dose given daily was able to increase the *K. pneumoniae* enrofloxacin MIC 8-fold (from 0.047 µg/mL to 0.38 µg/mL). Our findings suggest the need for studies to assess if the same could occur in humans.

**IMPORTANCE:**

Antimicrobial-resistant infections are responsible for over a million deaths a year. Reducing antimicrobial resistance requires addressing all the sources of unnecessary antimicrobial exposure. Because the antimicrobial concentration in our food frequently approaches or exceeds the maximum allowed limits, it is crucial to ensure that the legal concentrations of antimicrobials in food do not induce antimicrobial resistance. We found that enrofloxacin doses, 10 times lower than those classified as safe, could increase enrofloxacin MICs 8-fold in *K. pneumoniae* in the gastrointestinal tracts of mice. These findings suggest that we need to consider the induction of antimicrobial resistance when defining safe concentrations of antimicrobials in our food.

## INTRODUCTION

A growing body of evidence suggests that the concentrations of antimicrobials allowed in the food we eat may select for antimicrobial resistance (1). Over 10 years ago, Gullberg et al. provided evidence that antimicrobial concentrations more than 100 times lower than the minimal inhibitory concentration (MIC) can select for antimicrobial resistance (AMR) in *Escherichia coli* and *Salmonella enterica* spp. (2, 3). They defined the minimum concentration of an antimicrobial that is able to select for antimicrobial resistance as the minimum selective concentration (MSC) (2, 4). There are two types of MSC: the first is the minimum concentration of an antimicrobial that can induce *de novo* resistance (MSC_denovo_), and the second is the lowest antimicrobial concentration that selects for a resistant- compared with a susceptible-strain (MSC_select_) (2, 5). Gullberg et al. found the *E. coli* ciprofloxacin MSC_select_ to be 230-fold lower than the MIC, and the MSC_denovo_ to be at least 10-fold lower than the MIC. They did not assess if even lower concentrations could select for *de novo* resistance (4). More recent *in vitro* experiments have established that ciprofloxacin concentrations as low as 1/1,000th of the MIC can induce resistance in *Neisseria gonorrhoeae* (6) and *Neisseria subflava* (7). In the case of fosfomycin, concentrations 1/2,000th of the MIC can select for resistant mutants (8). A systematic review of 62 studies found that subinhibitory concentrations of fluoroquinolones were able to select for increases in MIC for a wide range of gram-positive and -negative organisms (9). The mechanisms responsible included both target mutations and enhanced efflux pumps (9).

The published ciprofloxacin MSCs of *E. coli*, *N. subflava,* and *N. gonorrhoeae* (0.1 to 0.004 µg/L) are considerably lower than the measured concentration of quinolones in meat, water, and environmental samples collected from a number of locations (2, 4, 6, 7). Studies from East Asia have, for example, reported mean concentrations of ciprofloxacin in edible fish to be 331.7 µg/kg (10). A recent study from the United Kingdom found that the median concentration of enrofloxacin in beef in London supermarkets was 533 µg/kg (11). Eating food with high quinolone concentrations has also been found to be associated with high urinary and fecal concentrations of quinolones in humans (12–15). A study from South Korea, for example, found high concentrations of enrofloxacin and ciprofloxacin in the urine of the general population and found that these were strongly associated with the consumption of beef, chicken, and dairy products  (12). Likewise, a study in northern China found that veterinary fluoroquinolones were detected in the urine of 57% of healthy children (16). Another study in China found ciprofloxacin and enrofloxacin concentrations above the level of detection in the faeces of 67% and 30% of individuals, respectively. The median concentration of fluoroquinolone detected was 20  µg/kg. Since enrofloxacin is used for food by animals and not by humans, the likely source of the enrofloxacin was the ingestion of veterinary antimicrobials in food  (13).  One study has found that eliminating meat consumption resulted in lower urinary fluoroquinolone concentrations  (17). Some studies have also found that the consumption of meat is a risk factor for AMR (18, 19). The Rotterdam Study, for example, found that ciprofloxacin resistance in community-acquired urinary tract infections was associated with a high intake of pork and chicken (20). 

Ecological studies from Europe and elsewhere have found positive associations between quinolone resistance in *E. coli* infections in humans and the total consumption of quinolones in animals (21–23). Importantly, studies have also shown that reducing antimicrobial use in food-producing animals can result in reduced AMR in humans  (24). These findings have led us and others to hypothesize that the low doses of residual fluoroquinolones allowed in food could select for AMR in our resident microbiota (1, 25, 26).

The European Medicines Agency (EMA) calculates the acceptable daily ingestion (ADI) of a medicinal compound based on studies evaluating thresholds for microbiological and cellular toxicity (25–30). The EMA fluoroquinolone ADIs are determined based on microbiological toxicity (30, 31). These are established by evaluating the MICs (but not the MSCs) of common human bacterial commensals/pathobionts such as *E. coli* based on estimated dose exposures in the human colon (25–27, 30). ADIs serve as the basis for determining maximum residue limits (MRLs), which specify the highest permissible concentration of a compound in food products, taking into account typical consumption patterns (27, 32).

The most recent EMA reports concluded that the acceptable daily intake of enrofloxacin is 372 µg for an average person of 60 kg (30). We have recently found that this dose (6.2 µg/kg) and one-tenth of this dose are able to select for *de novo* ciprofloxacin resistance in a *Galleria mellonella* model of chronic *Klebsiella pneumoniae* infection (33). In a similar vein, we found that an ADI dose of erythromycin was able to induce macrolide resistance in *Streptococcus pneumoniae* in a *G. mellonella* model (34). These experiments involved the injection of the target bacterium into the hemocoel of *G. mellonella* followed by the antimicrobial. It is possible that this model does not replicate the effect of orally ingested antimicrobials on the MICs of gastrointestinal bacteria in mammals (35). In the current study, we therefore assessed if ADI doses of enrofloxacin could induce enrofloxacin resistance in a murine model of chronic *K. pneumoniae* gut colonization. The mice received daily jellies containing ADI doses of enrofloxacin for 21 days. Fecal pellets were obtained from each mouse for culturing on selective agar, both with and without enrofloxacin to assess the emergence of elevated MICs to enrofloxacin.

## RESULTS

### Successful colonization with *K. pneumoniae*

**Fig 1:**
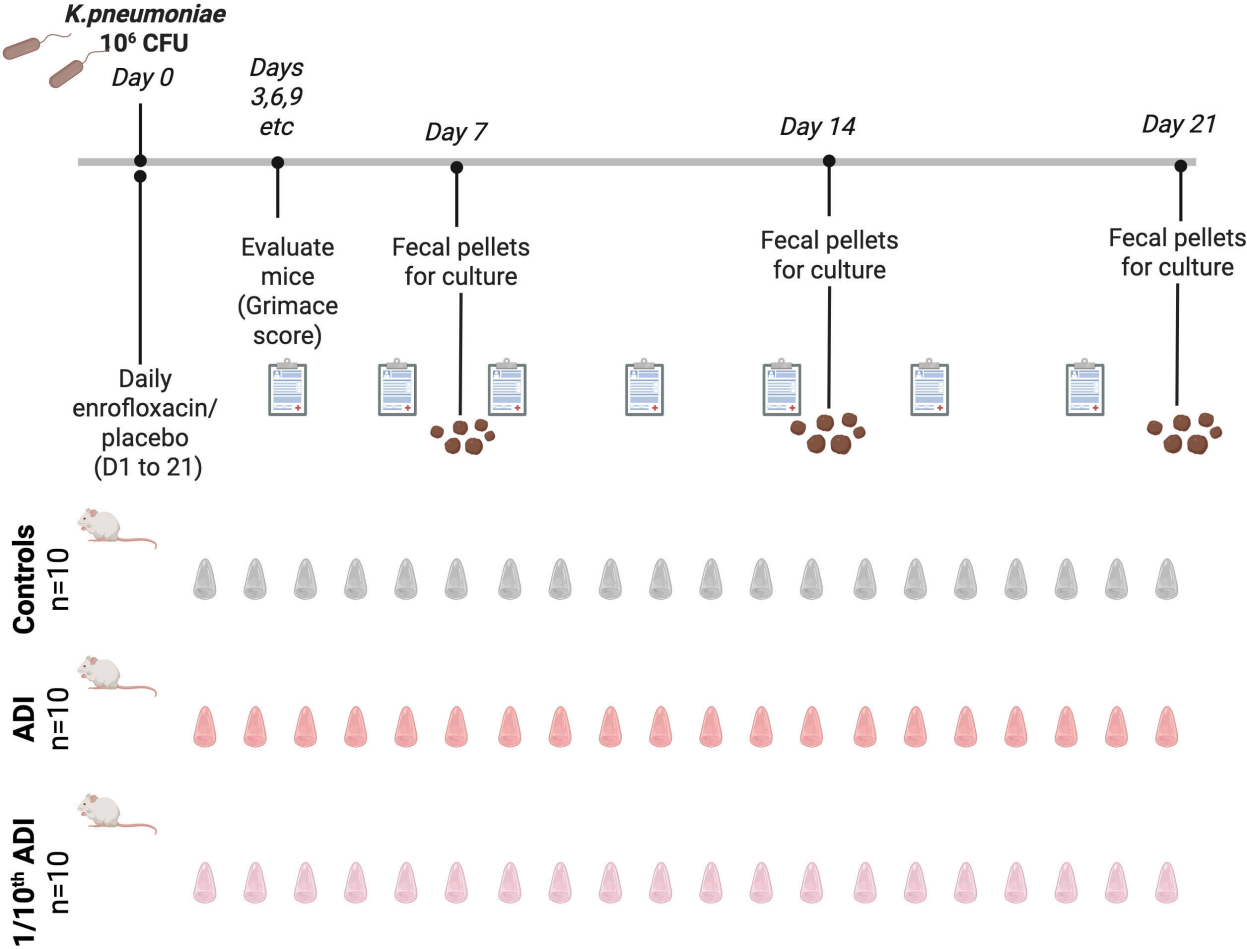
Schematic illustration of study methodology (Figure produced with BioRender).

Two to three mice per group of 10 mice were successfully colonized with *K. pneumoniae* (Table 1; Fig. 1). Colonization persisted for up to 21 days in the control and one-tenth ADI groups and for 14 days in the ADI group (Table 1).

**TABLE 1:** Colonization of mice with *K. pneumoniae* and changes in enrofloxacin MIC in the groups of mice exposed to enrofloxacin or placebo^*a*^

	Control						1/10xADI						ADI					
			No Ciprofloxacin		Ciprofloxacin				No Ciprofloxacin		Ciprofloxacin				No Ciprofloxacin		Ciprofloxacin	
Day	Mouse ID	Colony nr.	Colony count	Ciprofloxacin MIC	Colony count	Ciprofloxacin MIC	Mouse ID	Colony nr.	Colony count	Ciprofloxacin MIC	Colony count	Ciprofloxacin MIC	Mouse ID	Colony nr.	Colony count	Ciprofloxacin MIC	Colony count	Ciprofloxacin MIC
7	1	1	9	0.032	0	NA	1	1	19	0.032	4	0.047	3	1	8	0.032	2	0.38^*a*^
7	1	2		0.047			1	2		0.032		0.125^*a*^	3	2		0.032		0.064
7	1	3		0.047			1	3				0.047	5	1	37	0.047	46	0.25
7	1	4		0.032			1	4				0.064	5	2		0.047		0.19
7	1	5		0.032									5	3				0.047
7													5	4				0.38^*a*^
7							3	1	10	0.032	10	0.064	5	5				0.25
7							3	2		0.032		0.38^*a*^	6	1	2	0.047	0	NA
7							3	3				0.25^*a*^	6	2		0.032		
7							3	4				0.047						
7							3	5				0.064						
7																		
7																		
14	4	1	2	0.047	0	NA	1	1	24	0.047	40	0.047	5	1	10	0.047	16	0.064
14	4	2		0.047			1	2		0.047		0.064	5	2		0.047		0.38^*a*^
14							1	3				0.125^*a*^	5	3				0.047
14							1	4				0.064	5	4				0.064
14							1	5				0.094^*a*^	5	5				0.25
14							8	1	12	0.047	4	0.047						
14							8	2		0.047		0.064						
14							8	3				0.064						
14							8	4				0.047						
21	4	1	3	0.047	1	0.064^*a*^	1	1	63	0.047	69	0.094						
21	4	2		0.047			1	2		0.047		0.064						
21	4	3		0.047			1	3				0.032						
21							1	4				0.047						
21							1	5				0.032						
21							1	6				0.032						
21							1	7				0.064						
21							1	8				0.047						
21							1	9				0.125^*a*^						
21							1	10				0.064						

^*a*^ These isolates were sent for whole genome sequencing; NA- Not Applicable; ADI – Acceptable Daily Intake.

### Emergence of reduced susceptibility to enrofloxacin resistance in both ADI groups

By day 7, *K. pneumoniae* colonies with reduced susceptibility to enrofloxacin had emerged in both the ADI groups (Table 1). In the one-tenth ADI group, two mice were found to be colonized with *K. pneumoniae* at this time point. One colony from Mouse 1 had an enrofloxacin MIC of 0.125 µg/mL, and two colonies from Mouse 8 had elevated enrofloxacin MICs (0.25 µg/mL and 0.38 µg/mL; Table 1). In the ADI group, three mice were colonized with *K. pneumoniae* on day 7, and five *K. pneumoniae* colonies with reduced susceptibility to enrofloxacin were detected in two of these mice (mice 3 and 4 with MICs ranging between 0.19 µg/mL and 0.38 µg/mL; Table 1).

By day 14, *K. pneumoniae* was recovered from the same two mice in the one-tenth ADI group. Only two colonies of *K. pneumoniae* with reduced enrofloxacin susceptibility were detected from one of these mice (MICs 0.094 µg/mL and 0.125 µg/mL; Table 1). At this time point, one of the three mice in the ADI group that was colonized on day 7 was still colonized with *K. pneumoniae*, and two colonies with reduced susceptibility to enrofloxacin were isolated from this mouse (MICs 0.25 µg/mL and 0.38 µg/mL; Table 1).

At day 21, no mice in the ADI group and only one mouse in the one-tenth ADI group were still colonized with *K. pneumoniae*. Two colonies from this mouse had reduced susceptibility to *K. pneumoniae* (0.094 µg/mL and 0.125 µg/mL; Table 1).

In the control group, a single mouse was found to be colonized with *K. pneumoniae* on day 7, whereas another mouse was colonized on days 14 and 21, and no *K. pneumoniae* colonies with reduced susceptibility to enrofloxacin were detected in these mice (Table 1).

### Emergent resistance-associated mutations

All the isolates of *K. pneumoniae* recovered from the mice and sent for whole genome sequencing (WGS) were nearly identical to the baseline *K. pneumoniae* administered to each mouse (Sequence Type [ST] 220; Table 1). No canonical fluoroquinolone-associated mutations were detected (Table S4). A missense mutation (Asn288Ser) in the *nnr* gene encoding for bifunctional NAD(P)H-hydrate repair enzyme was identified in the one-tenth ADI group sample (colony number 5 of mouse 1 with a MIC of 0.094 µg/mL) (Table S4).

## DISCUSSION

We found that oral enrofloxacin doses as low as one-tenth of the dose classified by the EMA as safe increased the enrofloxacin MICs of gastrointestinal *K. pneumoniae*. These findings are very similar to those (7) found in the *G. mellonella* model colonized with the same strain of *K. pneumoniae* (36). In this previous study, we likewise found that one-tenth of the ADI dose of ciprofloxacin resulted in an 8-fold increase in ciprofloxacin MIC of *K. pneumoniae* M14827 (36). Similarly, resistance development following sub-MIC antibiotic exposure has been reported for other antibiotic classes, including macrolides, beta-lactams, and trimethoprim (7, 34, 37). For instance, previous *in vivo* studies have found that sub-ADI doses of erythromycin and trimethoprim could induce AMR in *S. pneumoniae* and *E. coli,* respectively (33, 34, 37).

The concentrations of fluoroquinolones in food frequently exceed the legal limits defined by the MRLs/ADIs. A systematic review of the topic found that antimicrobial residue concentration exceeded MRLs in 52 of the 73 studies reviewed (38). For example, in the survey of fluoroquinolones in beef in British supermarkets noted above, the median concentration of enrofloxacin in beef (533 mg/kg, IQR 235–1060) was over five times higher than the EMA MRL (100 mg/kg) (11). The highest enrofloxacin concentration (5,976 mg/kg) was found in chicken wings (11). Likewise, a study in poultry in Nigeria found that 46% of the samples analyzed had detectable enrofloxacin (mean concentration: 458 mg/kg) (39). These findings suggest that concentrations of fluoroquinolones exceed the MRLs in food in a number of countries. Moreover, the frequent detection of residues exceeding current MRLs may also reflect non-compliance with withdrawal periods, inadequate regulatory enforcement, or pharmacokinetic variability among animals (38). An additional pathway to consider is that antimicrobials in food could select for AMR in the microbes in food, which could then transfer their resistance mechanisms to bacteria in the human microbiome. A recent study found over 1,000 bacterial species in various foodstuffs, with 3% of these species being common constituents of the human microbiome (40). When considered in conjunction with results from previous MSC experiments (7, 34, 36), our study results suggest that the lowest concentrations of antimicrobials that can select for resistance should be included in the criteria used to define ADIs and MRLs.

Despite the strains showing an 8-fold increase in enrofloxacin MIC in the initial phenotypic assay, no enrofloxacin resistance-associated mutations were identified via WGS. Several factors may explain this discrepancy, including the potential instability of the resistance-conferring mutations and their loss over time due to selective pressures, laboratory handling, or storage conditions (41). One possible explanation is that the resistance-conferring mutations were unstable and could have been lost during the culturing process with the freezing, thawing, and re-streaking that may have contributed to this instability, especially in the absence of selective pressure from antibiotics. Some mutations are not stable and can be rapidly lost in the absence of selective pressure, such as during periods of growth without antibiotic exposure due to phenotypic reversion (41–43). It is thus possible that the resistance mutations were present initially but were not genetically stable. Over successive generations in the absence of antibiotic selection, these mutations might have been lost, leading to the detection of a susceptible genotype in WGS despite the initial high MIC phenotype. E-testing was repeated on the nine isolates after WGS revealed no resistance mutations. This found low enrofloxacin MICs. Similar findings have been reported in studies where strains with high MICs initially lose resistance markers after prolonged storage and culturing without antibiotic pressure (44). A recent study on the MSC select of enrofloxacin in broiler-derived cecal fermentations also failed to detect genotypic evidence of fluoroquinolone resistance. The study found an enrofloxacin MSC of around 0.00125 mg/L in this complex environment, but no difference in fluoroquinolone mutations or the abundance in quinolone-resistance genes (*qnr*) between the enrofloxacin-exposed and control groups (45). Studies have found that sub-MIC concentrations of fluoroquinolones can induce AMR via activation of the bacterial SOS response (46). The SOS response can result in AMR via induction of hypermutability, horizontal gene transfer, biofilm production, and bacterial tolerance (47, 48). Future work could investigate if any of these pathways are implicated in the emergence of AMR in the mouse model we used.

There are a number of other limitations to our study. We only evaluated the effect of a single antimicrobial on a single strain of a single target species. We were only able to evaluate two doses of this antimicrobial. We found that reduced susceptibility to enrofloxacin emerged at the lowest dose tested. We are, therefore, not able to assess if even lower doses would have had a similar effect. EUCAST defines ciprofloxacin resistance in *Enterobacterales* as >0.5 µg/mL (49). The highest enrofloxacin MICs in our experiment were below the threshold of 0.38 µg/mL. We defined the MSC conservatively as the lowest dose of enrofloxacin which resulted in a 2-fold increase in MIC. Based on this definition and our results, we can only state that the MSC in our experiment was one-tenth of the ADI dose of enrofloxacin or lower. Quite a low proportion of the mice were successfully colonized with *K. pneumoniae*, which made our sample size small. The proportion of colonized individuals is dependent on factors such as colonization resistance of the mice’s gastrointestinal tract microbiota (50), inoculum dose, or strain-specific colonization fitness (51). The mice from each condition were cohoused for the duration of the experiment, except for once a day when they were separated to receive their jelly. This means they may have transferred their resistant or susceptible *K. pneumoniae* to one another via contact or coprophagy. Our results are therefore best interpreted per condition and not per mouse. A number of the *K. pneumoniae* colonies growing on the enrofloxacin plates did not have elevated MICs. This finding is likely explained by the fact that the enrofloxacin concentration in the enrofloxacin plates was only 2.5-fold higher than the MIC. We did not evaluate for collateral resistance, but in our previous experiment to define the ciprofloxacin MSC for the same strain of *K. pneumoniae*, we found that ciprofloxacin induced cross-resistance to doxycycline and ceftriaxone (33). There are a number of adverse effects associated with low-dose antimicrobials, which we were unable to assess. For example, low doses of enrofloxacin (0.1–50 mg/L) have been found to increase the *Firmicutes* to *Bacteroidetes* ratio, which has been linked to obesity and inflammatory bowel disease (52, 53). Low-dose enrofloxacin has been found to increase the abundance of fluoroquinolone resistance-associated mutations in certain gut genera more than others (52). Sub-MIC concentrations of antimicrobials can also promote horizontal gene transfer and biofilm formation (54). We did not measure these effects or the concentration of enrofloxacin and other fluoroquinolones in the mouse food. However, if quinolones present in the food were responsible for the observed increase in enrofloxacin MICs, then we would expect to see elevated MICs emerging in the control group. This increase was not seen.

A possible next step in this research would be to ascertain if ADI doses of antimicrobials could induce resistance in the human microbiota. In the Healthy Food RCT, we randomized individual participants to either ADI doses of ciprofloxacin or placebo (EUCT number: 2023–506205-18-00). The primary outcome was the effect on the fecal *E. coli* ciprofloxacin MICs. The results of this study are expected in 2025. These results will enable us to evaluate if the *G. mellonella* or mouse model can be used as surrogates for evaluating ADIs/MRLs for human consumption. If the *G. mellonella* model is found to be adequate, then this would provide a cheap, high-throughput model to test the *in vivo* MSCs of various antimicrobial-bacteria combinations.

## MATERIALS AND METHODS

### Bacterial strain

We used the same *K. pneumoniae* M14827 strain from our previous MSC experiments in *G. mellonella* (33). This strain is a clinical isolate collected at the Institute of Tropical Medicine, Antwerp. This strain has an enrofloxacin and ciprofloxacin MIC of 0.047 µg/mL, as ascertained in triplicate by gradient diffusion assay (Etest) (33).

### Experimental groups

We used 48 to 54-day-old Naval Medical Research Institute (Han) Specific Opportunistic Pathogen-Free (SOPF) mice. Their SOPF status ensured that they were not pre-colonized with *Klebsiella pneumoniae*. Three experimental groups of 10 mice were used: (i) mice fed 1 ADI of enrofloxacin daily, (ii) mice fed one-tenth ADI of enrofloxacin daily, and (iii) mice fed no enrofloxacin (negative control group). These sample sizes were determined based on a pilot study involving five mice that were inoculated with the same number of CFUs of *K. pneumoniae* M14827. *K. pneumoniae* was successfully retrieved from the feces of two of the five mice over a period of 5 days. The ADI dose for the mice was calculated based on the mean weight of the mice (27.13 g; Table S1) and the EMA ADI: 6.2 µg/kg (30, 55).

#### Colonization of the mice

Our colonization protocol closely followed that of Young et al., who were able to successfully set up a chronic model of *K. pneumoniae* GIT colonization lasting at least 30 days (56).

*K. pneumoniae* M14827 was grown overnight at 37°C in 5% CO_2_ on Columbia agar plates supplemented with 5% sheep blood. A suspension of 10^7^ CFUs/100 µL in 2% sucralose-PBS was prepared to inoculate the mice (Table S3). Each mouse was orally administered 100 µL of the 10^7^ CFUs in 100 µL 2% sucralose-PBS using a thin dropper inserted at the corner of the mouth. All mice were individually marked on their tails for identification.

#### Administration of enrofloxacin

The day after receiving the *K. pneumoniae,* each mouse was given a jelly containing the appropriate dose of enrofloxacin. The jelly was flavored and prepared based on the protocol of Zhang (Table S2) (57). This method prevents the need for gavage and has been successfully validated in a number of studies (58, 59). The mice received this jelly daily for the entire study duration (21 days). The enrofloxacin concentration in the jellies was adjusted according to each group (one-tenth ADI and 1 ADI). The control group received a placebo jelly, which was identical in composition, except it did not contain any enrofloxacin.

#### Isolation of K. pneumoniae

To quantify bacterial shedding, we collected three fecal pellets from each individual mouse every 7 days during the course of the study. This was done by placing each mouse in a separate cage for 5 min (Fig. 1). The fecal pellets were collected in 1 mL screw-cap tubes, and 500 µL of PBS was added. The mixture was homogenized using a micro-pestle, vortexed, and spun down. A 100 µL aliquot of the supernatant was plated on a *K. pneumoniae* Chromoselect Selective Agar plate (Merck [Darmstadt, Germany]) both with (0.125 µg/mL) and without enrofloxacin. The agar plates were then incubated for 16–20 h.

#### *Determination of the MSC*_*de novo*_ *– in vivo*

After 16–20 h of incubation, all colonies were counted and then replated on Mueller-Hinton (MH) agar. Identification of all individual colonies per plate was performed via MALDI-TOF-MS (33, 34). In some instances, due to high colony density or colonies being too close together, it was not possible to isolate all individual colonies for replating. However, all colonies that were successfully replated and grew were subsequently identified via MALDI-TOF MS. Enrofloxacin MICs of up to five colonies per plate were determined via a gradient diffusion assay (Liofilchem, Roseto degli Abruzzi, Italy). In keeping with previous studies, we defined the MSC_de novo_ as the lowest concentration that resulted in a doubling of the enrofloxacin MIC (33, 34).

#### Storage of the samples

All isolates were stored in skim milk supplemented with 20% glycerol at −80°C.

### Species identification via MALDI-TOF MS

For species identification, each bacterial isolate was spread on a steel target plate and covered with 1 µL of 70% formic acid, followed by 1 µL of α-cyano-4-hydroxycinnamic acid (CHCA) matrix solution. The target plate was allowed to dry and then loaded and read. The spectra were acquired in linear mode within a mass range of 2–20 kDa and compared with a reference library. The results were classified as reliable or unreliable according to recommended cutoff values of 1.7 and 2 for validated results for the genus and species-level identifications, respectively. For further details, please refer to (33).

### Whole genome sequencing

WGS was conducted as necessary to evaluate putative resistance-associated mutations and confirm the identity of *K. pneumoniae* M14827. A random selection of nine *K. pneumoniae* isolates with the highest evolved enrofloxacin MICs and two controls was chosen for this (Table 1). The samples were outsourced to Eurofins (Germany), where total DNA was extracted. Library preparation was performed using a Stranded TruSeq DNA library preparation kit. Sequencing was conducted on the NextSeq 6000 platform, v2 with 2 × 150 bp (Illumina Inc., San Diego, CA, USA).

For the WGS analysis, initial quality control of the raw reads was carried out using FastQC (60). The raw reads were trimmed using Trimmomatic (v0.39) (Phred score 33 and length of the bases ≥ 30 bases) (61). The processed raw reads were *de novo*-assembled using SPAdes (v3.14.0) using the following parameters: trim—depth 150—opts—careful (62). The quality of the *de novo*-assembled contigs was evaluated using Quast (v5.0.2) (63) and checkM (64), followed by genome annotation using Prokka (v1.14.6) (65). The quality-controlled reads were mapped to respective reference draft genomes (No CIP group, day 21, mouse id 4, colony nr-3; Table 1), and single-nucleotide polymorphisms were identified using Snippy with default parameters (66). The raw reads generated are deposited at BioProject ID PRJNA1165977.

### Euthanasia

At the end of the experiment, the mice were euthanized using CO_2_.

### Classification of decreased susceptibility to enrofloxacin

A two-fold increase in enrofloxacin MIC was considered a relevant increase in MIC.

## Supplementary Material

Reviewer comments

## Data Availability

The supplementary online file can be found at: https://figshare.com/articles/dataset/Supplementary_online_file/28839383?file=53889035.

## SUPPLEMENTAL MATERIAL

OPEN PEER REVIEW reviewer-comments.pdf An accounting of the reviewer comments and feedback.

ASM does not own the copyrights to Supplemental Material that may be linked to, or accessed through, an article. The authors have granted ASM a non-exclusive, world-wide license to publish the Supplemental Material files. Please contact the corresponding author directly for reuse.
