# Supplementary material for: “Acceptable” concentrations of enrofloxacin in food lead to reduced enrofloxacin susceptibility in a mouse model of gastrointestinal Klebsiella pneumoniae
Source: Microbiol Spectr. 2025 May 22;13(7):e00385-25. doi: 10.1128/spectrum.00385-25 (PMC12210917; doi:10.1128/spectrum.00385-25)
Supplement: Reviewer comments [file reviewer-comments.pdf]

AMERICAN SOCIETY FOR MICROBIOLOGY | Microbiology Spectrum

'Acceptable' concentrations of enrofloxacin in food lead to reduced enrofloxacin susceptibility in a mouse model of gastrointestinal *Klebsiella pneumoniae*

Zina Gestels, Bianca Torfs, Said Abdellati, Irith De Baetselier, Caroline Rombouts, Veronique Dermauw, Sheeba Manoharan-Basil, and Christopher Kenyon

Corresponding Author(s): Christopher Kenyon, Instituut voor Tropische Geneeskunde

Review Timeline:

Submission Date:	February 7, 2025
Editorial Decision:	March 21, 2025
Revision Received:	April 8, 2025
Accepted:	April 14, 2025

Editor: Dhammika Navarathna

Reviewer(s): Disclosure of reviewer identity is with reference to reviewer comments included in decision letter(s). The following individuals involved in review of your submission have agreed to reveal their identity: Ákos Jerzsele (Reviewer #1)

Transaction Report:

(Note: With the exception of the correction of typographical or spelling errors that could be a source of ambiguity, letters and reports are not edited. The original formatting of letters and referee reports may not be reflected in this compilation.)

DOI: https://doi.org/10.1128/spectrum.00385-25

Re: Spectrum00385-25 (**'Acceptable' concentrations of enrofloxacin in food lead to reduced enrofloxacin susceptibility in a mouse model of gastrointestinal *Klebsiella pneumoniae***)

Dear Prof. Christopher Kenyon:

Thank you for the privilege of reviewing your work. Below you will find my comments, instructions from the Spectrum editorial office, and the reviewer comments.

Please return the manuscript within 60 days; if you cannot complete the modification within this time period, please contact me. If you do not wish to modify the manuscript and prefer to submit it to another journal, notify me immediately so that the manuscript may be formally withdrawn from consideration by Spectrum.

Revision Guidelines
To submit your modified manuscript, log into the submission site at https://spectrum.msubmit.net/cgi-bin/main.plex. Go to Author Tasks and click the appropriate manuscript title to begin. The information you entered when you first submitted the paper will be displayed; update this as necessary. Note the following requirements:

• Upload point-by-point responses to the issues raised by the reviewers in a file named "Response to Reviewers," NOT in your cover letter.
• Upload a compare copy of the manuscript (without figures) as a "Marked-Up Manuscript" file.
• Upload a clean .DOC/.DOCX version of the revised manuscript and remove the previous version.
• Each figure must be uploaded as a separate, editable, high-resolution file (TIFF or EPS preferred), and any multipanel figures must be assembled into one file.
• Any supplemental material intended for posting by ASM should be uploaded with their legends separate from the main manuscript. You can combine all supplemental material into one file (preferred) or split it into a maximum of 10 files with all associated legends included.

For complete guidelines on revision requirements, see our Submission and Review Process webpage. Submission of a paper that does not conform to guidelines may delay acceptance of your manuscript.

Data availability: ASM policy requires that data be available to the public upon online posting of the article, so please verify all links to sequence records, if present, and make sure that each number retrieves the full record of the data. If a new accession number is not linked or a link is broken, provide Spectrum production staff with the correct URL for the record. If the accession numbers for new data are not publicly accessible before the expected online posting of the article, publication may be delayed; please contact production staff (Spectrum@asmusa.org) immediately with the expected release date.

Publication Fees: For information on publication fees and which article types are subject to charges, visit our website. If your manuscript is accepted for publication and any fees apply, you will be contacted separately about payment during the production process; please follow the instructions in that e-mail. Arrangements for payment must be made before your article is published.

ASM Membership: Corresponding authors may join or renew ASM membership to obtain discounts on publication fees. Need to upgrade your membership level? Please contact Customer Service at Service@asmusa.org.

The ASM Journals program strives for constant improvement in our submission and publication process. Please tell us how we can improve your experience by taking this quick Author Survey.

Thank you for submitting your paper to Spectrum.

Sincerely,
Dhammika Navarathna
Editor
Microbiology Spectrum

Reviewer #1 (Comments for the Author):

This manuscript addresses a highly relevant and pressing issue, presenting compelling findings that highlight the potential risks posed by antibiotic residues in food, even when present at concentrations below the Acceptable Daily Intake (ADI) threshold. The study is well-designed, scientifically rigorous, and reproducible, providing important insights. Given its significance, further studies of this nature would be highly beneficial. However, several aspects merit further consideration:

1. It would be valuable to include a discussion on whether similar findings have been reported for other antibiotic classes. Are there comparable in vivo studies beyond the mentioned in vitro investigations? Additionally, do the authors plan to conduct similar experiments with different antibiotics and bacterial strains?
2. The observed colonization success rate appears low. How do the authors interpret this result? Do they consider it acceptable or suboptimal? What factors could underline this outcome - inoculum dose, strain virulence, or other variables?
3. How do the authors assess the fact that, in multiple instances, antibiotic residues were detected above the Maximum Residue Limit (MRL)? Does this suggest non-compliance with regulatory guidelines, or could it indicate that the officially established withdrawal periods may require reassessment? Alternatively, could there be other overlooked contributing factors?
4. The discussion on the absence of mutations during MIC elevation under very low antibiotic concentrations is well-structured and logically presented. However, have the authors considered the role of oxidative stress? Is there a possible threshold effect in this context? Furthermore, what about the SOS response genes - were they analyzed in this study?

Overall, this is a well-executed study with a robust experimental model, providing reliable and reproducible data. Addressing these questions - though optional - would help clarify important aspects of the study and enhance the discussion.

This manuscript addresses a highly relevant and pressing issue, presenting compelling findings that highlight the potential risks posed by antibiotic residues in food, even when present at concentrations below the Acceptable Daily Intake (ADI) threshold.

The study is well-designed, scientifically rigorous, and reproducible, providing important insights. Given its significance, further studies of this nature would be highly beneficial. However, several aspects merit further consideration:

1. It would be valuable to include a discussion on whether similar findings have been reported for other antibiotic classes. Are there comparable in vivo studies beyond the mentioned in vitro investigations? Additionally, do the authors plan to conduct similar experiments with different antibiotics and bacterial strains?

2. The observed colonization success rate appears low. How do the authors interpret this result? Do they consider it acceptable or suboptimal? What factors could underline this outcome — inoculum dose, strain virulence, or other variables?

3. How do the authors assess the fact that, in multiple instances, antibiotic residues were detected above the Maximum Residue Limit (MRL)? Does this suggest non-compliance with regulatory guidelines, or could it indicate that the officially established withdrawal periods may require reassessment? Alternatively, could there be other overlooked contributing factors?

4. The discussion on the absence of mutations during MIC elevation under very low antibiotic concentrations is well-structured and logically presented. However, have the authors considered the role of oxidative stress? Is there a possible threshold effect in this context? Furthermore, what about the SOS response genes — were they analyzed in this study?

Overall, this is a well-executed study with a robust experimental model, providing reliable and reproducible data. Addressing these questions — though optional — would help clarify important aspects of the study and enhance the discussion.

Dear Reviewer,

We thank you for your encouraging comments, which have helped us to further strengthen the manuscript. Below, we provide a point-by-point response to the best of our knowledge and indicate where modifications have been incorporated into the manuscript.

Reviewer #1 (Comments for the Author):

This manuscript addresses a highly relevant and pressing issue, presenting compelling findings that highlight the potential risks posed by antibiotic residues in food, even when present at concentrations below the Acceptable Daily Intake (ADI) threshold.
The study is well-designed, scientifically rigorous, and reproducible, providing important insights. Given its significance, further studies of this nature would be highly beneficial.
However, several aspects merit further consideration:
1. It would be valuable to include a discussion on whether similar findings have been reported for other antibiotic classes. Are there comparable in vivo studies beyond the mentioned in vitro investigations? Additionally, do the authors plan to conduct similar experiments with different antibiotics and bacterial strains?

Response: We have now added text to the discussion (lines 185-189) noting that similar findings have been reported for other antibiotic classes, including macrolides, nitrofurantoin and beta-lactams. In particular, we reference studies demonstrating that ADI concentrations of erythromycin selected for macrolide resistance in *Streptococcus pneumoniae* using a *Galleria mellonella* model. We also cite a recent *in vivo* study which found that sub-ADI concentrations of trimethoprim were able to induce trimethoprim resistance in *Escherichia coli*. Regarding, follow-up experiments involving additional antibiotic classes and bacterial species-we did perform a RCT study in human volunteers comparing ADI doses of ciprofloxacin with a placebo on changes in ciprofloxacin MICs on gut *E. coli* MICs that we have now referred to in the discussion in lines 272-276.

Lines 185-189 Similarly, resistance development following sub-MIC antibiotic exposure has been reported for other antibiotic classes, including macrolides, beta-lactams and trimethoprim (36, 38, 39). For instance, previous in vivo studies have found that sub ADI doses of erythromycin and trimethoprim could induce AMR in *S. pneumoniae,* and *E. coli,* respectively (1, 2).

Lines 268-270. “If the *G. mellonella* model is found to be adequate, then this would provide a cheap, high-throughput model to test the *in vivo* MSCs of various antimicrobial-bacteria combinations”.

2. The observed colonization success rate appears low. How do the authors interpret this result? Do they consider it acceptable or suboptimal? What factors could underline this outcome - inoculum dose, strain virulence, or other variables?

Response: Indeed, the colonization rate was lower than expected and is mentioned in discussion lines (238-241) likely arising due to a combination of factors, including the colonization resistance provided by the murine gut microbiota, potential variability in microbiota composition across animals, and possibly suboptimal colonization potential of the strain used.

We have adapted the text as follows:

Lines 238-241: Quite a low proportion of the mice were successfully colonized with *K. pneumoniae*, which made our sample size small. The proportion colonized is dependent on factors such as colonization resistance of the mice's gastrointestinal tract microbiota (3), inoculum dose or strain-specific colonization fitness (4).

3. How do the authors assess the fact that, in multiple instances, antibiotic residues were detected above the Maximum Residue Limit (MRL)? Does this suggest non-compliance with regulatory guidelines, or could it indicate that the officially established withdrawal periods may require reassessment? Alternatively, could there be other overlooked contributing factors?

Response: We agree with the reviewer that all these factors could play a role and have now expanded the discussion to address this in lines 200-202.

Lines 200-202: Moreover, the frequent detection of residues exceeding current MRLs may also reflect non-compliance with withdrawal periods, inadequate regulatory enforcement, or pharmacokinetic variability among animals (5).

4. The discussion on the absence of mutations during MIC elevation under very low antibiotic concentrations is well-structured and logically presented. However, have the authors considered the role of oxidative stress? Is there a possible threshold effect in this context? Furthermore, what about the SOS response genes - were they analyzed in this study?
Overall, this is a well-executed study with a robust experimental model, providing reliable and reproducible data. Addressing these questions - though optional - would help clarify important aspects of the study and enhance the discussion.

Response: Although our study did not assess oxidative stress pathways or the SOS response in detail, we agree these are plausible mechanisms and could be explored in future studies, possibly also using transcriptomic approaches. We have now included this to lines 230-232.

Lines 230-232. Studies have found that sub-MIC concentrations of fluoroquinolones can induce AMR via activation of the bacterial SOS response (49). The SOS response can result in AMR via induction of hypermutability, horizontal gene transfer, biofilm production and bacterial tolerance (50, 51). Future work could investigate if any of these pathways are implicated in the emergence of AMR in the mouse model we used.

References

1. Macleod JK, Gestels Z, Abdellati S, Vanbaelen T, Kenyon C, Manoharan-Basil SS. 2024. Determination of the De Novo Minimum Selection Concentration of Trimethoprim In Vivo for Escherichia coli Using Galleria mellonella: A Pilot Study. Microorganisms 13.

2. Baranchyk Y, Gestels Z, Van den Bossche D, Abdellati S, Britto Xavier B, Manoharan-Basil SS, Kenyon C. 2024. Effect of erythromycin residuals in food on the development of resistance in *Streptococcus pneumoniae* : an *in vivo* study in *Galleria mellonella*. PeerJ 12:e17463.

3. Buffie CG, Pamer EG. 2013. Microbiota-mediated colonization resistance against intestinal pathogens. Nat Rev Immunol 13:790–801.

4. Cheung BH, Alisoltani A, Kochan TJ, Lebrun-Corbin M, Nozick SH, Axline CMR, Bachta KER, Ozer EA, Hauser AR. 2023. Genome-wide screens reveal shared and strain-specific genes that facilitate enteric colonization by *Klebsiella pneumoniae*. mBio 14.

5. Treiber FM, Beranek-Knauer H. 2021. Antimicrobial Residues in Food from Animal Origin—A Review of the Literature Focusing on Products Collected in Stores and Markets Worldwide. Antibiotics 10:534.

6. Bush NG, Diez-Santos I, Sankara Krishna P, Clavijo B, Maxwell A. 2025. Insights into antibiotic resistance promoted by quinolone exposure. Antimicrob Agents Chemother 69.

7. Memar MY, Yekani M, Celenza G, Poortahmasebi V, Naghili B, Bellio P, Baghi HB. 2020. The central role of the SOS DNA repair system in antibiotics resistance: A new target for a new infectious treatment strategy. Life Sci 262:118562.

Re: Spectrum00385-25R1 (**'Acceptable' concentrations of enrofloxacin in food lead to reduced enrofloxacin susceptibility in a mouse model of gastrointestinal *Klebsiella pneumoniae***)

Dear Prof. Christopher Kenyon:

Your manuscript has been accepted, and I am forwarding it to the ASM production staff for publication. Your paper will first be checked to make sure all elements meet the technical requirements. ASM staff will contact you if anything needs to be revised before copyediting and production can begin. Otherwise, you will be notified when your proofs are ready to be viewed.

Data Availability: ASM policy requires that data be available to the public upon online posting of the article, so please verify all links to sequence records, if present, and make sure that each number retrieves the full record of the data. If a new accession number is not linked or a link is broken, provide production staff with the correct URL for the record. If the accession numbers for new data are not publicly accessible before the expected online posting of the article, publication may be delayed; please contact ASM production staff immediately with the expected release date.

Publication Fees: For information on publication fees and which article types have charges, please visit our website. We have partnered with Copyright Clearance Center (CCC) to collect author charges. If fees apply to your paper, you will receive a message from no-reply@copyright.com with further instructions. For questions related to paying charges through RightsLink, please contact CCC at ASM_Support@copyright.com or toll free at +1-877-622-5543. CCC makes every attempt to respond to all emails within 24 hours.

ASM Membership: Corresponding authors may join or renew ASM membership to obtain discounts on publication fees. Need to upgrade your membership level? Please contact Customer Service at Service@asmusa.org.

PubMed Central: ASM deposits all Spectrum articles in PubMed Central and international PubMed Central-like repositories immediately after publication. Thus, your article is automatically in compliance with the NIH access mandate. If your work was supported by a funding agency that has public access requirements like those of the NIH (e.g., the Wellcome Trust), you may post your article in a similar public access site, but we ask that you specify that the release date be no earlier than the date of publication on the Spectrum website.

Embargo Policy: A press release may be issued as soon as the manuscript is posted on the Spectrum Latest Articles webpage. The corresponding author will receive an email with the subject line "ASM Journals Author Services Notification" when the article is available online.

The ASM Journals program strives for constant improvement in our submission and publication process. Please tell us how we can improve your experience by taking this quick Author Survey.

Thank you for submitting your paper to Spectrum.

Sincerely,
Dhammika Navarathna
Editor
Microbiology Spectrum
